# The Free Lunch is not over yet—systematic exploration of numerical thresholds in maximum likelihood phylogenetic inference

**DOI:** 10.1093/bioadv/vbad124

**Published:** 2023-09-14

**Authors:** Julia Haag, Lukas Hübner, Alexey M Kozlov, Alexandros Stamatakis

**Affiliations:** Computational Molecular Evolution Group, Heidelberg Institute for Theoretical Studies, 69118 Heidelberg, Germany; Computational Molecular Evolution Group, Heidelberg Institute for Theoretical Studies, 69118 Heidelberg, Germany; Institute for Theoretical Informatics, Karlsruhe Institute of Technology, 76131 Karlsruhe, Germany; Computational Molecular Evolution Group, Heidelberg Institute for Theoretical Studies, 69118 Heidelberg, Germany; Computational Molecular Evolution Group, Heidelberg Institute for Theoretical Studies, 69118 Heidelberg, Germany; Institute for Theoretical Informatics, Karlsruhe Institute of Technology, 76131 Karlsruhe, Germany; Biodiversity Computing Group, Institute of Computer Science, Foundation for Research and Technology – Hellas, 70013 Heraklion, Greece

## Abstract

**Summary:**

Maximum likelihood (ML) is a widely used phylogenetic inference method. ML implementations heavily rely on numerical optimization routines that use internal numerical thresholds to determine convergence. We systematically analyze the impact of these threshold settings on the log-likelihood and runtimes for ML tree inferences with RAxML-NG, IQ-TREE, and FastTree on empirical datasets. We provide empirical evidence that we can substantially accelerate tree inferences with RAxML-NG and IQ-TREE by changing the default values of two such numerical thresholds. At the same time, altering these settings does not significantly impact the quality of the inferred trees. We further show that increasing both thresholds accelerates the RAxML-NG bootstrap without influencing the resulting support values. For RAxML-NG, increasing the likelihood thresholds $\epsilon_{\text{LnL}}$ and $\epsilon_{\text{brlen}}$ to 10 and 10^3^, respectively, results in an average tree inference speedup of 1.9 ± 0.6 on *Data collection 1*, 1.8 ± 1.1 on *Data collection 2*, and 1.9 ± 0.8 on *Data collection 2* for the RAxML-NG bootstrap compared to the runtime under the current default setting. Increasing the likelihood threshold $\epsilon_{\text{LnL}}$ to 10 in IQ-TREE results in an average tree inference speedup of 1.3 ± 0.4 on *Data collection 1* and 1.3 ± 0.9 on *Data collection 2*.

**Availability and implementation:**

All MSAs we used for our analyses, as well as all results, are available for download at https://cme.h-its.org/exelixis/material/freeLunch_data.tar.gz. Our data generation scripts are available at https://github.com/tschuelia/ml-numerical-analysis.

## 1 Introduction

Phylogenetic trees have many important applications in biology and medicine, e.g. in drug development (Gregoretti *et al.* 2004), forensics (Metzker *et al.* 2002), or the analysis of SARS-CoV-2 genomes (Morel *et al.* 2021). A widely used approach for reconstructing phylogenetic trees from a multiple sequence alignment (MSA) is the maximum likelihood (ML) method (Yang *et al.* 1995). Popular ML-based tools are RAxML-NG (Kozlov *et al.* 2019), IQ-TREE (Minh *et al.* 2020), and FastTree (Price *et al.* 2010). Finding the most likely tree is NP-hard (Chor and Tuller 2005) due to the super-exponential number of possible tree topologies. ML tree inference tools therefore implement tree search heuristics that attempt to iteratively optimize the log-likelihood (LnL score) by improving the tree topology, branch lengths, and substitution model parameters. These heuristics heavily rely on a plethora of numerical optimization routines [e.g. typically Brent’s method (Brent 1971) and the Broyden-Fletcher-Goldfarb-Shanno (BFGS) method (Fletcher 2000)] that use specific internal numerical convergence thresholds. To the best of our knowledge, the impact of these threshold settings on inference times and LnL scores has never been systematically assessed, while anecdotal observations do exist. For instance, when analyzing SARS-CoV-2 data, Morel *et al.* (2021) observed that one of these numerical thresholds, the minimum allowed branch length (*minBranchLen*), impacts the LnL scores of trees inferred with RAxML-NG and IQ-TREE. Here, we systematically investigate if we can reproduce this effect on other MSAs as well as for additional numerical thresholds. In addition to RAxML-NG and IQ-TREE, we also investigate the behavior of FastTree. We explore the influence of up to seven distinct numerical thresholds on LnL scores and runtimes for these three ML inference tools. For each tool, we analyze the influence of these settings on the standard tree inference procedure. During this standard tree inference procedure, the tools strive to optimize the tree topology, the branch lengths, and the substitution model parameters based on an initial starting tree topology. RAxML-NG and IQ-Tree also offer a tree evaluation procedure. During this tree evaluation procedure, the given (user-defined) tree topology remains fixed while only the branch lengths and substitution model parameters are being optimized. For RAxML-NG and IQ-Tree, we also analyze the influence of the numerical thresholds on this tree evaluation procedure.

A frequently used execution flavor in RAxML-NG is the ––all mode. In addition to inferring 20 ML trees, RAxML-NG infers bootstrap replicate trees, and draws the bootstrap support values onto the best-scoring out of the 20 inferred ML trees. By default, RAxML-NG infers at most 1000 bootstrap replicates, but implements an early-stopping criterion that determines convergence based on the bootstopping criterion introduced by Pattengale *et al.* (2010). For RAxML-NG, we also analyze the influence of two likelihood epsilon thresholds on the results and the runtime of the bootstrapping procedure.

Our analyses comprise four main studies. The following paragraph summarizes each study and highlights the most important results.

**Study 1:** In this first exploratory study, we analyzed the influence of up to seven numerical thresholds on the LnL scores and runtimes of the RAxML-NG, IQ-TREE, and FastTree tree inference procedures. For RAxML-NG and IQ-TREE, we also analyzed the influence of the same thresholds when varied during the tree evaluation procedure. In this first study, we exclusively analyzed unpartitioned empirical DNA MSAs (*Data collection 1*). We observe a substantial runtime impact on tree inferences for two likelihood epsilons in RAxML-NG ($\epsilon_{\text{LnL}}$ and $\epsilon_{\text{brlen}}$). We further find that we can increase the settings of both thresholds without compromising the quality of the inferred trees, while obtaining a speedup of 1.9 ± 0.6. We make a similar observation for one numerical threshold ($\epsilon_{\text{LnL}}$) in IQ-TREE that yields a speedup of 1.3 ± 0.4. All other thresholds we analyzed for RAxML-NG and IQ-TREE, as well as all thresholds analyzed for FastTree do not substantially influence neither runtime nor LnL scores as long as these settings remain within a reasonable range. For all analyzed ML inference tools, their current default settings fall within this reasonable range. As expected, we observe that the runtime of the evaluation phase is small compared to the corresponding tree inference time. Despite the impact of some numerical thresholds on tree evaluation runtimes, we therefore recommend using a conservative numerical threshold setting for tree evaluation.

**Study 2:** To verify the findings of *Study 1* for the likelihood epsilons $\epsilon_{\text{LnL}}$ and $\epsilon_{\text{brlen}}$ in RAxML-NG, and $\epsilon_{\text{LnL}}$ in IQ-TREE, we subsequently analyzed a more comprehensive as well as representative collection of empirical MSAs, including DNA, amino-acid (AA), and partitioned MSAs (*Data collection 2*). Our analyses on this more comprehensive data collection confirm our observations regarding tree inferences: the speedup for RAxML-NG is 1.8 ± 1.1 and 1.3 ± 0.9 for IQ-TREE. Analogous to our results on *Data collection 1*, we do not observe a significant impact on the quality of the inferred trees according to our evaluation metrics.

**Study 3:** In our third study, based on the results of *Study 2*, we analyze the impact of the $\epsilon_{\text{LnL}}$ and $\epsilon_{\text{brlen}}$ thresholds on the RAxML-NG bootstrapping procedure. *Study 2* suggests that both thresholds can be increased for tree inferences without compromising the quality of the inferred trees, yet resulting in faster analyses. The hypothesis is that we can safely increase both thresholds to accelerate bootstrapping as well. We test this hypothesis using the MSAs of *Data collection 2*. Our analyses suggest that both likelihood epsilon settings can be increased without compromising the bootstrapping results and yield a speedup of 1.9 ± 0.8 for *Data collection 2*.

**Study 4:** In our final study, we conducted a more detailed analysis of the likelihood epsilons in RAxML-NG as it is being actively developed in our lab. Since RAxML-NG uses the same threshold $\epsilon_{\text{LnL}}$ for four distinct operations during its tree inference procedure, we separated this threshold into four distinct fine-grained likelihood epsilons. The goal was to assess if appropriate fine-grained threshold settings further improve runtimes. Our analyses suggest that separating the $\epsilon_{\text{LnL}}$ into four distinct thresholds does not further improve runtimes. We observe a similar behavior for all four thresholds, both in terms of tree inference quality and runtime. We hence conclude that such a fine-grained distinction of threshold settings is neither necessary nor beneficial.

The remainder of this article is organized as follows: In Section 2, we outline the numerical thresholds we analyze and their usage in ML inference tools, our experimental setup, and the metrics we used to assess the influence of the numerical thresholds on tree inference quality, bootstrapping quality, and runtime. In Section 3, we present our key findings and results of our analyses. To limit the extent of this article, we only describe and discuss the results of *Study 2* and *Study 3* in greater detail. The results of *Study 1* and *Study 4* are available in the Supplementary Material.

All MSAs we used for our analyses, as well as all results, are available for download at https://cme.h-its.org/exelixis/material/freeLunch_data.tar.gz. Our data generation scripts are available at https://github.com/tschuelia/ml-numerical-analysis.

## 2 Methods

### 2.1 Numerical thresholds

Due to the extremely large tree space, an exhaustive search to identify the most likely tree is not feasible. ML-based tree inference tools therefore typically implement iterative tree improvement techniques, which they apply to an initial (starting) tree. Such an initial topology is obtained via heuristic tree inference methods [e.g. randomized stepwise addition order (Cavalli-Sforza and Edwards 1967) or maximum parsimony (Farris 1970, Fitch 1971)]. In our analyses, we focus on the three widely used ML inference tools RAxML-NG, IQ-TREE, and FastTree. Each tool iteratively optimizes the tree topology, the branch lengths, and the substitution model parameters starting from an initial tree. For example, RAxML-NG iteratively applies Subtree Pruning and Regrafting (SPR) moves followed by branch length and substitution model parameter optimizations. We provide a more detailed description of the tree search heuristics in Supplementary Section 1. In our initial exploratory study *Study 1* we analyze the influence of the following seven numerical thresholds:

- Likelihood epsilon $\epsilon_{\text{LnL}}$: Threshold for LnL score improvement after one complete iteration (tree topology, branch lengths, and model parameters). The optimization only continues if the likelihood improvement is higher than this threshold.
- Branch length likelihood epsilon $\epsilon_{\text{brlen}}$: RAxML-NG specific threshold for LnL score improvement. This epsilon is used during a so-called fast branch length optimization to rapidly approximate the LnL score of potential SPR moves.
- Minimum branch length (*minBranchLen*): Lower limit for branch length values.
- Maximum branch length (*maxBranchLen*): Upper limit for branch length values.
- Model likelihood epsilon $\epsilon_{\text{model}}$: Threshold for substitution model parameter improvement. The substitution model parameters are only further optimized if the LnL score improvement exceeds this threshold.
- *num_iters*: Threshold to control the maximum number of iterations during Newton-Raphson based branch length optimization in RAxML-NG.
- *bfgs_factor*: This RAxML-NG specific threshold controls the convergence of the L-BFGS-B method used for optimizing substitution rates and stationary frequencies. The L-BFGS-B is a variant of the standard BFGS method, optimized for limited memory, and is extended to incorporate bound constraints in variables (Zhu *et al.* 1997).

For RAxML-NG we analyze the influence of all seven thresholds, for IQ-TREE we analyze the influence of $\epsilon_{\text{LnL}}$, *minBranchLen*, *maxBranchLen*, and $\epsilon_{\text{model}}$. For FastTree we analyze the influence of $\epsilon_{\text{LnL}}$ and *minBranchLen*. Table 1 shows a comprehensive list of the threshold settings we analyze. In all follow-up studies (*Studies 2–4*) we focus on the following thresholds: $\epsilon_{\text{LnL}}$ and $\epsilon_{\text{brlen}}$. In *Study 1* we find that decreasing the default settings does not substantially improve the LnL scores. To economize on computational resources and runtime, we thus only compare the current default setting to larger settings in *Studies 2–4*. For IQ-TREE, the current default setting for $\epsilon_{\text{LnL}}$ is $10^{-3}$. We analyze the potentially more liberal/superficial settings $\left\{10^{-3},10^{-2},\ldots ,10^{3}\right\}$. For RAxML-NG the current default setting for both, $\epsilon_{\text{LnL}}$ and $\epsilon_{\text{brlen}}$, is $10^{-1}$. We analyze more superficial settings of $\left\{10^{-1},1,\ldots ,10^{3}\right\}$

**Table 1.** Numerical thresholds we varied, including the analyzed settings and respective inference tools where they are applicable.^a^

Threshold	Tested settings	Inference tools (resp. default setting)
*minBranchLen*	{10^–10^,10^–9^, …, 10^–2^}^b^	RAxML-NG ($10^{-6}$)
		IQ-TREE ($10^{-6}$)
		FastTree ($5^{-9}$)
*maxBranchLen*	$\left\{10,10^{2}\right\}$	RAxML-NG (10^2^)
		IQ-TREE (10)
$\epsilon_{\text{LnL}}$	$\left\{10^{-3},10^{-2},\ldots ,10^{3}\right\}$	RAxML-NG ($10^{-1}$)
		IQ-TREE ($10^{-3}$)
		FastTree ($10^{-1}$)
$\epsilon_{\text{model}}$	$\left\{10^{-3},10^{-2},10^{-1}\right\}$	RAxML-NG ($10^{-3}$)
		IQ-TREE ($10^{-2}$)
$\epsilon_{\text{brlen}}$	$\left\{10^{-3},10^{-2},\ldots ,10^{3}\right\}$	RAxML-NG ($10^{-1}$)
*num_iters*	{16, 32, 64}	RAxML-NG (32)
*bfgs_factor*	$\left\{10^{5},10^{7},10^{9}\right\}$	RAxML-NG (10^7^)

a The values in parentheses indicate the default setting for the respective inference tool.

b For FastTree we additionally analyze its default setting $5^{-9}$.

### 2.2 Data collections

In our exploratory *Study 1* we analyze 22 empirical unpartitioned DNA MSAs (*Data collection 1*). For all follow-up studies (*Studies 2–4*), we analyze a broader collection of 19 empirical MSAs, including AA and partitioned MSAs (*Data collection 2*). For one additional AA dataset with excessive memory and runtime requirements, we only compare the results of the default threshold settings to the suggested new default settings. We exclusively analyze empirical datasets, because it was shown that reconstructing the best tree is more difficult on empirical datasets than it is on simulated datasets (Huelsenbeck 1995). Supplementary Section 2 provides a detailed overview of all MSAs we used for our analyses.

### 2.3 Experimental setup

In this section, we describe the experimental setup of our analyses. We separate this section into two paragraphs. In the first paragraph, we describe our experiments for analyzing the influence of the numerical thresholds on the tree inference and tree evaluation procedures (*Studies 1, 2, 4*). In the second paragraph, we describe our experiments for analyzing the influence of the likelihood epsilons on the bootstrapping procedure in RAxML-NG (*Study 3*). A more detailed description of our setup, as well as the software we use, is available in Supplementary Section 1.

#### 2.3.1 Tree inference and tree evaluation

We analyze each threshold and each ML inference tool separately. For each threshold and for each possible threshold setting, we infer 50 trees using the standard/default tree inference mode of the respective tool. Subsequently, we re-evaluate each inferred tree using the tree evaluation mode. During the tree evaluation, we set the numerical thresholds to their corresponding default values. In the set comprising *all* inferred trees under *all* analyzed threshold settings, we determine the tree with the best LnL score (henceforth referred to as *best-known tree*) and compare it to all other trees using several distinct phylogenetic statistical significance tests. For reasons, we detail further below, we do not compare all trees at once, but always conduct a pairwise comparison of each tree with the best-known tree. We collect trees that pass *all* significance tests in a so-called plausible tree set [see Morel *et al.* (2021) for the introduction of the term]. All trees in such a plausible tree set are not significantly worse than the best-known tree under all statistical significance tests.

To analyze the influence of the numerical thresholds on the RAxML-NG and IQ-Tree tree evaluation procedures, we use an analogous setup. Of the above-described pipeline, we reuse the 50 trees inferred under the current default setting of the respective numerical threshold. For each possible threshold setting, we re-evaluate each of the 50 trees using the tree evaluation mode and the respective threshold setting. The subsequent plausible tree set analysis is analogous to the setup for the tree inference procedure described above.

#### 2.3.2 Bootstrapping

In *Study 3* we exclusively analyze the influence of the likelihood epsilons on the RAxML-NG bootstrapping procedure. Since the bootstrapping procedure is computationally expensive, we refrain from testing all possible $\epsilon_{\text{LnL}}$ and $\epsilon_{\text{brlen}}$ settings in contrast to Studies 1, 2, and 4. Based on our findings in *Study 2* (see Section 3), we only compare the current default settings $\epsilon_{\text{LnL}}=0.1$ and $\epsilon_{\text{brlen}}=0.1$ to our suggested new settings $\epsilon_{\text{LnL}}=10$ and $\epsilon_{\text{brlen}}=10^{3}$. To compare the bootstrap results under both settings, we first infer 20 ML trees using RAxML-NG’s standard tree inference procedure. Based on our findings of *Study 2* (see Section 3), we set the likelihood epsilons to the suggested new settings $\epsilon_{\text{LnL}}=10$ and $\epsilon_{\text{brlen}}=10^{3}$ during this tree inference procedure. For both parameter configurations [(0.1, 0.1), (10, 10^3^)], we separately infer bootstrap replicates using RAxML-NG and map the bootstrap support values onto all 20 inferred ML trees.

#### 2.3.3 Model of evolution

For all experiments described above, we set the substitution model according to the following rules: For the unpartitioned DNA MSAs we use the general time reversible (GTR) model (Tavaré 1986) of nucleotide substitution as it is a flexible and general model of nucleotide substitution that is widely used and computationally efficient (Sumner *et al.* 2012). To account for among site rate heterogeneity, we also use four discrete Γ rate categories. The AA equivalent of the GTR model is the GTR20 (or PROTGTR) model. However, this model for AA data is very parameter rich. In particular, on datasets with weak phylogenetic signal (see below) the corresponding parameter estimates might thus be unstable. Instead, we use the LG substitution model (Le and Gascuel 2008) with four discrete Γ rate categories for unpartitioned AA MSAs. For partitioned MSAs, we use the partition file as provided alongside the MSA in the respective data source (see Supplementary Material).

### 2.4 Evaluation metrics

#### 2.4.1 Tree inference and tree evaluation

In the following, we compare LnL scores in percent rather than via absolute LnL unit difference, since the datasets cover a broad range of absolute LnL values [LnL scores range between approximately –90 (D4) and –13 000 000 (D37)]. Thus, as LnL scores are reported on a log scale, the observed effects are greater than the percentages might suggest. Therefore, we use two additional quality metrics: statistical significance tests and Robinson-Foulds distances (RF-Distances) (Robinson and Foulds 1981) which we describe further below. While our plausible tree set analyses do incorporate the branch length estimates to some extent, as the statistical tests are based on the ML scores of the trees, we additionally analyze the impact of the suggested changes on the branch lengths using the K Tree Score (KTS). For evaluating the runtimes of the tree inferences, we compute the speedup by comparing the runtime of each tree inference in relation to the average runtime under the respective default setting. We report all speedups as mean ± standard deviation. To ensure fair comparisons, we use identical hardware for all per-dataset experiments. Additionally, for the tree inference experiments, we fix the random seed to ensure that tree inferences always initiate their search on the same starting tree, despite using different numerical threshold settings. Note that in our analyses we do not compare inferred trees, LnL scores, runtimes, or evaluation metrics across ML inference tools. All described analyses and evaluations metrics are applied separately and independently to each tool.

##### 2.4.1.1 Significance tests

In order to compare the trees inferred under different threshold settings, we use the statistical significance tests implemented in IQ-TREE. IQ-TREE implements the following significance tests: the Kishino–Hasegawa (KH) test (Kishino and Hasegawa 1989) and the Shimodaira–Hasegawa (SH) test (Shimodaira and Hasegawa 1999), both in their weighted and unweighted variants, the Approximately Unbiased (AU) test (Shimodaira 2002), as well as the Expected Likelihood Weight (ELW) test (Strimmer and Rambaut 2002). We use the default IQ-TREE settings for the number of resampling of estimated log-likelihoods (RELL) replicates (10 000) and the significance level (α=0.05). We further denote a tree passing all statistical tests when compared to the best-known tree as being *plausible*. As described above, we collect all plausible trees per threshold setting in a plausible tree set. In subsequent analyses, we also use the number of plausible trees per setting, i.e. the size of the respective plausible tree sets, as well as the number of unique plausible tree topologies ($N_{\text{pl}}$) and their average pairwise RF-Distance ($RF_{\text{pl}}$). Since the significance tests can be biased by the number of trees in the candidate set (Strimmer and Rambaut 2002), we remove identical tree topologies from the set of inferred trees prior to applying the tests. Despite this tree set cleaning, we observed some unexpected behavior by the significance tests. First, the ELW test computes a c-ELW score (posterior weight) for each tree, sorts the trees according to this score and accepts trees as being not significantly different until the sum of c-ELW scores exceeds a predefined threshold. In our case, numerous trees in the inferred tree set have highly similar LnL scores despite their topologies being different. Yet, the c-ELW score for such trees is identical. Therefore, for trees that have a c-ELW that is close to exceeding the predefined significance threshold, only some trees with the exact same c-ELW score are accepted while the remaining ones are rejected. This leads to trees being rejected despite having identical LnL score as some accepted trees. Further, re-running the significance tests with the same trees but in a different order leads to a different subset of trees being accepted. Instead of re-estimating the substitution model parameters of each candidate tree, IQ-TREE uses a given best tree to optimize these parameters and uses them for all other trees. As stated above, numerous trees have identical LnL scores, and therefore choosing the best tree according to the LnL score is ambiguous. We observe that the results of the significance tests vary largely depending on what tree is passed as the best tree, despite identical LnL scores. We provide an example for both scenarios in the Supplementary Information. For the above reasons, instead of comparing all trees in the inferred tree set to each other at once, we only compare each inferred tree separately via all significance tests in a pairwise manner to the best-known tree. However, the c-ELW test is not intended for pairwise comparisons and only rejects one of the trees if the LnL scores deviate largely. Therefore, we also use the RF-Distance metric, which we describe in the following section.

##### 2.4.1.2 RF-distances

For the tree inference experiments, we fix the random seed to ensure that tree inferences always initiate their search on the same starting tree, despite using different numerical threshold settings. Therefore, we can directly compare tree topologies inferred under different numerical threshold settings that started on the same starting tree. We compare these trees in a pairwise manner via the relative RF-Distance. If the RF-Distance between two trees, e.g. one inferred under $\epsilon_{\text{LnL}}$ = $10^{-1}$ and one inferred under $\epsilon_{\text{LnL}}$ = 10^3^ is 0.0, then the tree inference converged to the same topology despite the different $\epsilon_{\text{LnL}}$ setting. However, an RF-Distance > 0 does not necessarily indicate that the tree is worse. For example, the plausible tree set generally comprises multiple distinct tree topologies which are not distinguishable via statistical significance tests. Therefore, when using this metric, we further compare these RF-Distances to the average pairwise RF-Distance between all plausible trees inferred under the default numerical threshold setting per tool (*default plausible trees*). We further refer to this RF-Distance as *default RF-Distance*. This *default RF-Distance* provides a notion of how topologically scattered the plausible trees are under the default numerical threshold settings. The higher the *default RF-Distance* is, the more rugged the tree space will be. If the *default RF-Distance* is greater or equal to the RF-Distance between trees inferred under different numerical threshold settings, we assume that these differences are due to the ruggedness of the tree space rather than the trees being worse.

##### 2.4.1.3 K tree score

In addition to analyzing the impact on the inferred tree topologies, we examine the influence of changing the threshold settings on the branch lengths. We follow a similar approach as described in the previous paragraph. We compare trees inferred using identical seeds but distinct threshold settings using the K tree score (KTS) (Soria-Carrasco *et al.* 2007), and examine the average KTS per MSA. The KTS is a normalized variant of the Branch Score Distance (Kuhner and Felsenstein 1994), where K refers to the scaling factor such that both trees have a similar global divergence rate. As baseline reference, we compute the average KTS between all plausible trees inferred under the default numerical threshold setting per tool (*default KTS*).

#### 2.4.2 Bootstrapping

To determine the influence of the likelihood epsilon settings on the quality of the RAxML-NG bootstrapping procedure, we adopt an analogous quality assessment strategy as Stamatakis *et al.* (2008). As described in Section 2.3, we infer 20 ML trees per MSA. For each of these 20 ML trees, we compare the bootstrap support values drawn onto those 20 trees based on bootstrap replicates inferred under the current default setting ($\epsilon_{\text{LnL}}=\epsilon_{\text{brlen}}=0.1$) to the support values drawn onto the same 20 trees based on bootstrap replicates inferred under the suggested new setting $\epsilon_{\text{LnL}}=10$ and $\epsilon_{\text{brlen}}=10^{3}$. Since we draw bootstrap support values on the same 20 ML trees, we can directly compare the values on a branch-by-branch basis using the Pearson correlation. This correlation only quantifies the relative similarity across bootstrap values. Hence, we also quantify the absolute difference between support values. To this end, we compute the pairwise absolute difference between support values under the old versus the suggested new setting across all branches of all 20 ML trees. Since RAxML-NG implements a bootstopping procedure (Pattengale *et al.* 2010), the number of bootstrap replicates may differ between likelihood epsilon configurations. We therefore also compare the number of computed replicates. We further summarize all bootstrap replicates per likelihood epsilon configuration in a consensus tree and compare the consensus trees between configurations using the RF-Distance. In the following analyses, we denote the consensus tree of all replicates inferred under $\epsilon_{\text{LnL}}=\epsilon_{\text{brlen}}=0.1$ as $C_{\text{default}}$ and the consensus for all replicates inferred under the suggested new settings (10, 10^3^) as $C_{\text{new}}$.

#### 2.4.3 Phylogenetic signal

The properties of the MSA influence the phylogenetic inference (Stamatakis 2011). The stronger the so-called *phylogenetic signal* in the data is, the easier the phylogenetic analysis will be. This phylogenetic signal provides a notion of how informative the data is about the underlying evolutionary process (Lemey *et al.* 2009). In our study, we use the sites-per-taxa ratio as a proxy for the phylogenetic signal. The sites-per-taxa ratio is computed by dividing the number of sites by the number of taxa in the MSA. In general, the higher the sites-per-taxa ratio of the MSA, the better the phylogenetic signal of the data will be. In the following analyses, we will refer to MSAs with a sites-per-taxa ratio ≥80 as *good phylogenetic signal*. We refer to MSAs with a lower sites-per-taxa ratio as MSA with an *intermediate* or *weak phylogenetic signal*. We are aware that quantifying this signal is a challenging task, and a plethora of alternative, more elaborate methods than the sites-per-taxa ratio have been proposed in the literature [see e.g. Misof *et al.* (2014) for an overview]. However, our analyses suggest that the impact of the numerical thresholds is well predicted by the sites-per-taxa ratio of the MSA under study, justifying our selection of this easy to compute proxy.

## 3 Discussion

In the following discussion, we focus on the analysis of the influence of $\epsilon_{\text{LnL}}$ and $\epsilon_{\text{brlen}}$ on the RAxML-NG and IQ-TREE tree inference (*Study 2*), as well as the influence of both thresholds on the RAxML-NG bootstrapping procedure (*Study 3*). All findings apply to *Data collection 2*. We discuss the less interesting results of *Study 1* and *Study 4* in detail in the Supplementary Material (Sections 3 and 5). The threshold with the highest impact on the runtimes of the RAxML-NG and IQ-Tree tree inference procedures is the likelihood epsilon $\epsilon_{\text{LnL}}$. We further observe a substantial impact of the branch length likelihood epsilon $\epsilon_{\text{brlen}}$ on the runtime of the RAxML-NG tree inference. Our analyses suggest that increasing these likelihood epsilon settings for RAxML-NG and IQ-TREE leads to equally good results, requiring less CPU time. The same observation holds true for the RAxML-NG bootstrapping procedure. All figures in the following section show the results summarized over all MSAs of *Data collection 2*. If not stated otherwise, we removed outliers using Tukey’s fences (Tukey 1977) with k:=3 for all figures depicting a speedup for better visualization. For the sake of completeness, we provide comprehensive speedup figures including all outliers in Supplementary Section 4. In all box plots, a dashed vertical line indicates the mean, and a solid vertical line the median value.

In the following, we discuss our analysis results for *Study 2* and *Study 3* on *Data collection 2*. In the first paragraph, we focus on the influence of the likelihood epsilons on tree inferences with RAxML-NG and IQ-Tree (*Study 2*). In the second paragraph, we present our results for the RAxML-NG bootstrapping procedure (*Study 3*).

### 3.1 Study 2: Tree inference

#### 3.1.1 RAxML-NG

**Figure 1.**
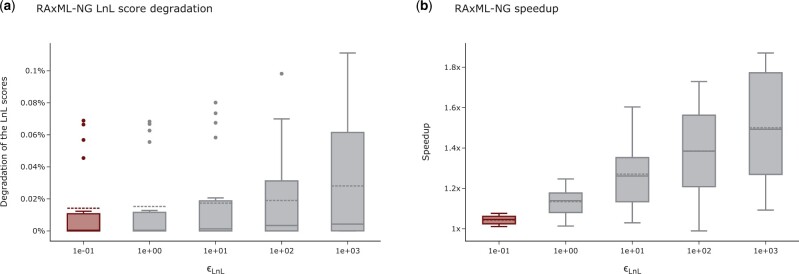
Influence of the $\epsilon_{\text{LnL}}$ setting on the LnL scores and runtime of RAxML-NG tree inferences. (a) Influence of the $\epsilon_{\text{LnL}}$ setting on the LnL scores of RAxML-NG. The highlighted box indicates the default setting. The *y*-axis shows the LnL score degradation per inferred tree in percent relative to the LnL score of the best-known tree. Higher percentages indicate worse LnL scores. (b) Influence of the $\epsilon_{\text{LnL}}$ setting on the RAxML-NG tree inference runtimes. The highlighted box indicates the default setting. The *y*-axis shows the speedup relative to the average runtime under the default setting.

With increasing $\epsilon_{\text{LnL}}$ threshold in RAxML-NG, we observe an expected decrease in LnL scores for higher settings. Especially for $\epsilon_{\text{LnL}}$ settings $\geq 10^{2}$ the LnL scores deteriorate noticeably (Fig. 1a). This is reflected by the proportion of tree inferences yielding a tree that is included in the plausible tree set (henceforth called a plausible tree) as well. For the RAxML-NG default setting $\epsilon_{\text{LnL}}=10^{-1}$ on average 85% of tree inferences yield a plausible tree, for 10^3^ on average only 83% yield a plausible tree. Averaged across all datasets, the $RF_{\text{pl}}$ increases from 0.13 ($10^{-1}$) to 0.16 (10^3^), and $N_{\text{pl}}$ from 17.4 to 24.6. For all datasets (except D15) the RF-Distances between trees inferred under $\epsilon_{\text{LnL}}\leq 10$ compared to the default setting $\epsilon_{\text{LnL}}=10^{-1}$ are smaller or equal to the *default RF-Distance*. However, for settings of 10^2^ and 10^3^ this is not the case. The average RF-Distances between trees inferred under these settings compared to the default setting are higher than the *default RF-Distance*. The topological differences among trees inferred under settings of 10^2^ and 10^3^ to trees inferred under the current default setting $10^{-1}$ can therefore not only be explained by the rugged tree space alone. This observation holds true even for datasets with a good phylogenetic signal. We conclude that for $\epsilon_{\text{LnL}}$ settings $\geq 10^{2}$ RAxML-NG infers worse trees than for settings below 10^2^. The runtimes of RAxML-NG tree inferences decrease with higher $\epsilon_{\text{LnL}}$ settings (Fig. 1b). On average, tree inferences under $\epsilon_{\text{LnL}}=10^{3}$ run approximately twice as fast as tree inferences under $\epsilon_{\text{LnL}}=10^{-1}$.

Given these observations, we conclude that the $\epsilon_{\text{LnL}}$ setting can be increased to 10. The quality of the trees is not affected by this more superficial optimization, but the tree inferences run on average 1.4 ± 0.6 times faster.

**Figure 2.**
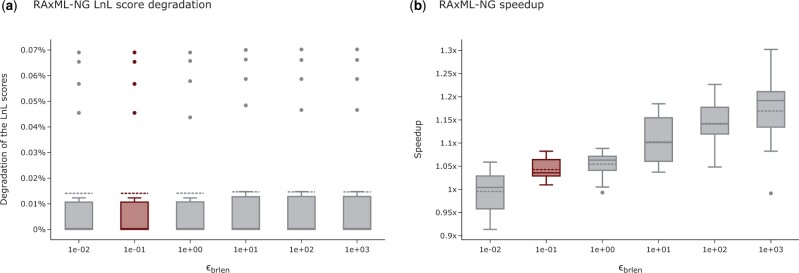
Influence of the $\epsilon_{\text{brlen}}$ setting on the LnL scores and runtime of RAxML-NG tree inferences. (a) Influence of the $\epsilon_{\text{brlen}}$ setting on the LnL scores of RAxML-NG. The highlighted box indicates the default setting. The *y*-axis shows the LnL score degradation per inferred tree in percent relative to the LnL score of the best-known tree. Higher percentages indicate worse LnL scores. (b) Influence of the $\epsilon_{\text{brlen}}$ setting on the RAxML-NG tree inference runtimes. The highlighted box indicates the default setting. The *y*-axis shows the speedup relative to the average runtime under the default setting.

With RAxML-NG, we also analyze the influence of the $\epsilon_{\text{brlen}}$ threshold. Similar to the $\epsilon_{\text{LnL}}$ threshold, the runtimes for $\epsilon_{\text{brlen}}$ improve with increasing settings (Fig. 2b). According to our analyses, the LnL score is unaffected by the $\epsilon_{\text{brlen}}$ setting (variations between settings ≤ 0.007%; Fig. 2a). Across all MSAs the number of tree inferences yielding a plausible tree is identical for all $\epsilon_{\text{brlen}}$ settings we analyze. The $RF_{\text{pl}}$ increases only slightly from 0.13 ($\epsilon_{\text{brlen}}=10^{-1}$) to 0.14 ($\epsilon_{\text{brlen}}=10^{-1}$). In analogy, $N_{\text{pl}}$ increases only slightly from 17.4 to 17.8 averaged over all datasets. For MSAs with a good phylogenetic signal, we observe that the $\epsilon_{\text{brlen}}$ setting does not affect the final tree topology: for all tested settings, the inferred tree topologies are identical (RF-Distance = 0.0). For all other MSAs, the average RF-Distance between trees inferred under different settings is below the *default RF-Distance*. We conclude that the $\epsilon_{\text{brlen}}$ threshold does not substantially influence the tree inference in RAxML-NG and the $\epsilon_{\text{brlen}}$ setting can be increased to 10^3^. In our analyses this observation holds true for all analyzed MSAs independently of the magnitude of the LnL scores. RAxML-NG uses the $\epsilon_{\text{brlen}}$ to optimize the three branch lengths that are adjacent to the node at which a subtree is regrafted via an SPR move. We suspect that since all branch lengths are optimized at a later step during the tree inference, conducting a thorough optimization of these three branch lengths does not substantially improve the LnL score and can thus be terminated early.

**Figure 3.**
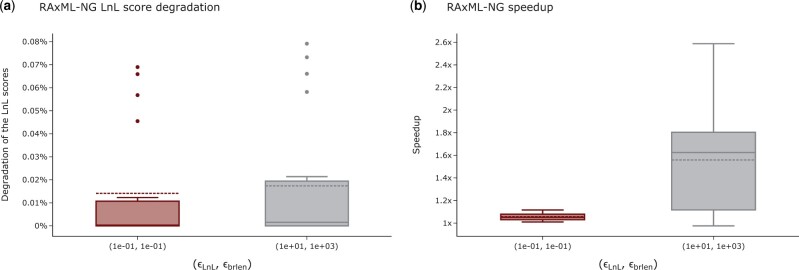
Influence of simultaneously changing both likelihood epsilon settings on the LnL scores and runtime of the RAxML-NG tree inference. (a) Influence of simultaneously changing both likelihood epsilon settings on the LnL scores of RAxML-NG. The highlighted box indicates the default combination. The *y*-axis shows the LnL score degradation per inferred tree in percent relative to the LnL score of the best-known tree. Higher percentages indicate worse LnL scores. (b) Influence of simultaneously changing both likelihood epsilon settings on the RAxML-NG tree inference runtimes. The highlighted box indicates the default combination. The *y*-axis shows the speedup relative to the average runtime under the default combination.

Since we suggest changing two likelihood epsilons in RAxML-NG, we further analyze the influence of simultaneously changing both settings on the quality and the runtimes of tree inferences. To limit the computational effort, we only compare the default combination $\left(\epsilon_{\text{LnL}},\epsilon_{\text{brlen}}\right)=(10^{-1},10^{-1})$ with the suggested new combination $\left(\epsilon_{\text{LnL}},\epsilon_{\text{brlen}}\right)=(10,10^{3})$. As expected, the LnL scores are worse under the new setting compared to the old setting (Fig. 3a), but the tree inferences are faster (Fig. 3b). Averaged over all MSAs, the LnL scores between the current default and the suggested new combination vary by <0.004%. For 16 out of 19 MSAs, the KTS between trees inferred under the current default combination versus the new combination are smaller or equal to the *default KTS*. For D815, we observe an unusually long branch of length 46 in one tree inferred under the current default setting, resulting in a KTS greater than the *default KTS*. This is likely to be a bug in the numerical branch length optimization procedure of RAxML-NG which we are currently investigating. Removing this single tree *prior* to averaging yields a KTS that is smaller than the *default KTS*. For D101 we observe a minor KTS increase: the *default KTS* is 0.2 while the KTS for trees inferred under the current default combination versus the new combination is 0.24. We observe a similar behavior for D140: the *default KTS* is 0.01 while the KTS for trees inferred under the current default combination versus the new combination is 0.09. We suspect that this is an artifact of the weak signal of both MSAs: the sites-per-taxa ratio is 18 for D101 and 8 for D140. The percentage of tree inferences yielding a plausible tree is identical under both setting combinations (87%). We observe only a minor increase of $RF_{\text{pl}}$ from 0.12 to 0.14 and $N_{\text{pl}}$ from 17.2 to 21.4. For all MSAs the RF-Distances between trees inferred under the current default combination versus the new combination are smaller or equal to the *default RF-Distance*. We conclude that increasing both threshold settings does not substantially decrease the LnL scores of the inferred trees and does therefore not affect the quality of the inferred trees. With the MSAs of *Dataset collection 1* we observe a speedup of 1.9 ± 0.6, on *Data collection 2* we observe a speedup of 1.8 ± 1.1.

#### 3.1.2 IQ-TREE

**Figure 4.**
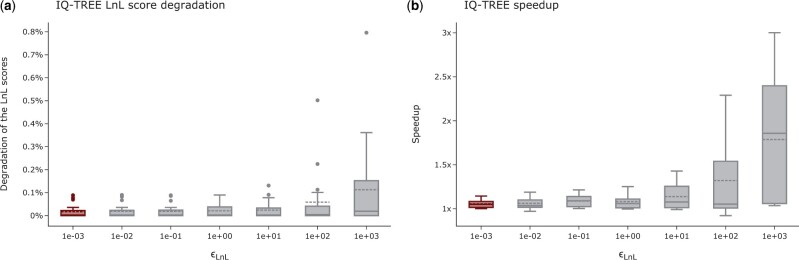
Influence of the $\epsilon_{\text{LnL}}$ setting on the LnL scores and runtimes of IQ-TREE tree inferences. (a) Influence of the $\epsilon_{\text{LnL}}$ setting on the LnL scores of IQ-TREE. The highlighted box indicates the default setting. The *y*-axis shows the LnL score degradation per inferred tree in percent relative to the LnL score of the best-known tree. Higher percentages indicate worse LnL scores. (b) Influence of the $\epsilon_{\text{LnL}}$ setting on IQ-TREE tree inference runtimes. The highlighted box indicates the default setting. The *y*-axis shows the speedup relative to the average runtime under the default setting.

Analogous to RAxML-NG, the runtimes of tree inferences improve with higher $\epsilon_{\text{LnL}}$ settings for IQ-Tree. Tree searches under the default setting of $\epsilon_{\text{LnL}}=10^{-3}$ run on average approximately twice as long as tree searches with $\epsilon_{\text{LnL}}=10^{3}$ (Fig. 4b). However, IQ-TREE appears to be more sensitive to the $\epsilon_{\text{LnL}}$ setting than RAxML-NG in terms of LnL scores. Under higher $\epsilon_{\text{LnL}}$ settings, the LnL score degradation is an order of magnitude worse than for RAxML-NG (on average ≤ 0.2% for IQ-TREE versus ≤ 0.03% for RAxML-NG; Fig. 4a). For $\epsilon_{\text{LnL}}$ values ≤10 the LnL scores are on average approximately equal. Also, based on the plausible tree set size under various settings, we observe that IQ-TREE is more sensitive to the $\epsilon_{\text{LnL}}$ setting. We observe that for $\epsilon_{\text{LnL}}=10^{3}$ averaged over all MSAs, noticeably fewer tree inferences yield a plausible tree than for any other setting (58% versus 76% for $\epsilon_{\text{LnL}}=10^{-3}$). This effect is less pronounced for MSAs with good phylogenetic signal. For MSAs with a sites-per-taxa ratio ≥80 we observe that the $\epsilon_{\text{LnL}}$ setting does not affect the final tree topology: under all tested settings the inferred tree topologies are identical (RF-Distance = 0.0). For MSAs with a worse phylogenetic signal, the RF-Distance between trees inferred under the default setting $10^{-3}$ and settings of 10^2^ and 10^3^ exceed the average RF-Distance in the plausible tree set. We conclude that for MSAs with an intermediate or weak phylogenetic signal, the trees inferred under $\epsilon_{\text{LnL}}$ settings $\geq 10^{2}$ are worse than under lower settings. According to our evaluation metrics across all analyzed MSAs the $\epsilon_{\text{LnL}}$ setting can be set to 10 without compromising the quality of the inferred tree topologies. For 17 out of 19 MSAs, the KTS between trees inferred under the current default setting versus the new setting are smaller or equal to the *default KTS*. For D80 we observe a minor increase in KTS: the *default KTS* is 0.17 while the KTS for trees inferred under the current default combination versus the new combination is 0.37. We observe a similar behavior for D815: the *default KTS* is 0.19 while the KTS for trees inferred under the current default combination versus the new combination is 0.36. We suspect that this is an artifact of the weak signal of both MSAs: the sites-per-taxa ratio is 25 for D815 and 3 for D80. In our analyses, the suggested increase of $\epsilon_{\text{LnL}}$ to 10 results in an average speedup of 1.3 ± 0.9.

As mentioned before, we observe a higher sensitivity to the $\epsilon_{\text{LnL}}$ setting in IQ-TREE than in RAxML-NG. We suspect that this is caused by the random Nearest Neighbor Interchange (NNI) topology perturbation moves in IQ-TREE’s search algorithm. IQ-TREE implements these random NNI moves to escape local NNI maxima (see the Supplementary Information for a more detailed description of the IQ-TREE inference heuristic). To explore this hypothesis, we modify IQ-TREE and disable this randomness in the search algorithm. As a consequence, IQ-TREE then only optimizes the tree topology using standard NNI moves. We refer to the standard IQ-TREE as *random IQ-TREE* and to the IQ-TREE algorithm without random NNI moves as *de-randomized IQ-TREE*. We re-analyze four MSAs using the de-randomized IQ-TREE version. Without the random NNI moves, the IQ-TREE search heuristic can explore the tree space less, thus, we expect the LnL scores for de-randomized IQ-TREE to be worse than for random IQ-TREE, which we indeed observe in our analyses. To compare the influence of the $\epsilon_{\text{LnL}}$ threshold, we again compute the proportion of tree inferences yielding a plausible tree. We observed that when using de-randomized IQ-TREE, noticeably more tree inferences yield a plausible tree under $\epsilon_{\text{LnL}}\geq 10^{2}$ than when using the random IQ-TREE variant. We conclude that large $\epsilon_{\text{LnL}}$ settings ($\geq 10^{2}$) distort the random NNI moves in IQ-TREE, causing a premature termination of the tree inference. This also explains the vast runtime improvement under these settings.

### 3.2 Study 3: RAxML-NG bootstrapping

Note that in the following discussion, we will refrain from reporting average correlation coefficients, as the most accurate method of averaging correlations is disputed (Corey *et al.* 1998). Especially, the widely used method of applying the Fisher z-transformation (Fisher 1992) prior to averaging is not directly applicable to our analyses results, as the inverse hyperbolic tangent function is only defined for values < 1.0. We do, however, frequently observe Pearson correlation coefficients of *exactly* 1.0.

**Figure 5.**
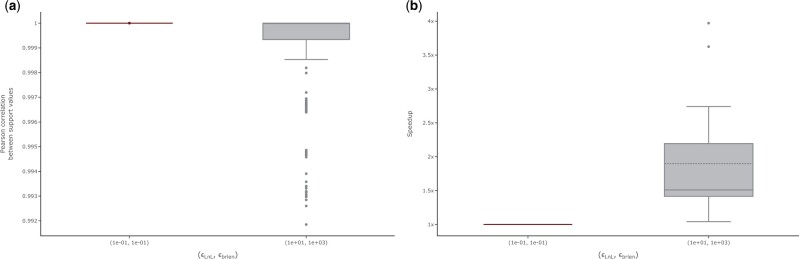
Influence of simultaneously changing both likelihood epsilon settings on the bootstrap support values and runtime of the RAxML-NG bootstrap. (a) Influence of simultaneously changing both likelihood epsilon settings on the bootstrap support values. The highlighted box indicates the default combination. The *y*-axis shows the Pearson correlation coefficients between support values for all ML trees across all analyzed datasets. (b) Influence of simultaneously changing both likelihood epsilon settings on the RAxML-NG bootstrapping runtimes. The highlighted box indicates the default combination. The *y*-axis shows the speedup relative to the runtime under the default combination. This figure shows all MSAs (no outlier filtering).

We observe Pearson correlation coefficients > 0.99 for all 20 ML trees when comparing the support values under the current default setting $\epsilon_{\text{LnL}}=\epsilon_{\text{brlen}}:=0.1$ to the suggested new setting $\epsilon_{\text{LnL}}:=10$ and $\epsilon_{\text{brlen}}:=10^{3}$ (Fig. 5a). All *P*-values are $\leq 10^{-35}$. We observe the lowest correlation coefficient on D354 (0.992). This is, however, not surprising, as this dataset contains highly similar ITS sequences, and is known to be difficulty to analyze (Grimm *et al.* 2006). RAxML-NG reports support values in percent on a scale of 0%–100%. Averaged over all datasets, the absolute pairwise difference in support values per tree topology is 0.5 percentage points. The highest observed difference is 3.23 percentage points for a phylogeny inferred on D25. On D25, the average support value over all 20 ML trees is 55.2%. This suggests that the bootstrap replicates inferred under both settings do not substantially influence the interpretation of the resulting support values drawn on the ML trees. As mentioned above, due to the implemented bootstopping procedure, the number of bootstrap replicates may differ depending on the likelihood epsilon setting. Except for two datasets, the computed number of bootstrap replicates is identical. For D354, we observe a slower convergence with the suggested new settings: RAxML-NG infers 700 replicates under (0.1,0.1) and 800 under $\left(10,10^{3}\right)$. In contrast, for D140, we observe a faster convergence with 400 inferred replicates under (0.1,0.1) versus 350 replicates under $\left(10,10^{3}\right)$. To further analyze this observation for D140 and D354, we reran the analyses using distinct random seeds. For D354, changing the random starting seed to 42 and 100 in distinct analyses, showed a similar trend: for seed = 42 the more conservative setting (0.1,0.1) converges after 850 bootstrap replicates while it does not converge under $\left(10,10^{3}\right)$ and infers the maximum number of 1000 replicates. With seed = 100 we observe 700 replicates under (0.1,0.1) versus 900 replicates under $\left(10,10^{3}\right)$. Yet, despite the increased number of inferred replicates, the increased likelihood epsilon settings result in a speedup > 1 compared to (0.1,0.1). For D140, changing the random starting seed from 0 to 42 resulted in identical number of replicates under both likelihood epsilon configurations (384 replicates).

Averaged over all MSAs, the relative RF-Distance between the respective bootstrap consensus trees $C_{\text{default}}$ and $C_{\text{new}}$ is 0.03. For 11 out of 20 MSAs, the consensus trees $C_{\text{default}}$ and $C_{\text{new}}$ are topologically identical (RF-Distance = 0.0). We observe the highest topological difference again for D354 (RF-Distance = 0.14). We conclude that the bootstrapping procedure is not affected by the increased likelihood epsilon values, and we suggest changing the respective default settings in RAxML-NG. Implementing the suggested changes results in a speedup of 1.9 ± 0.8 on *Data collection 2* (Fig. 5b).

## 4 Conclusion

Increasing the RAxML-NG settings for the likelihood epsilons $\epsilon_{\text{LnL}}$ and $\epsilon_{\text{brlen}}$ to 10 and 10^3^, respectively, does not significantly influence the quality of the inferred trees according to statistical significance tests. By changing both settings, we observe a speedup of 1.9 ± 0.6 on *Data collection 1* and 1.8 ± 1.1 on *Data collection 2*. With IQ-TREE, increasing the $\epsilon_{\text{LnL}}$ to 10 has no significant impact on the LnL scores, and we observe a speedup of 1.3 ± 0.4 on *Data collection 1* and 1.3 ± 0.9 on *Data collection 2*. Our observations are independent of the magnitude of the LnL scores of the analyzed MSAs. For MSAs with a good phylogenetic signal, the inferred tree topologies under the current default settings and the suggested new settings are identical for both, RAxML-NG and IQ-TREE (RF-Distance = 0.0). For MSAs with an intermediate or weak phylogenetic signal, the topological differences between threshold settings can be explained by the rugged tree space, and the RF-Distances between inferred trees under different settings are less than or equal to the *default RF-Distance*. It is important to note that the final tree evaluation after tree inference should not be omitted and performed under conservative likelihood epsilon settings, e.g. the default settings in RAxML-NG and IQ-TREE.

We further suggest increasing the $\epsilon_{\text{LnL}}$ and $\epsilon_{\text{brlen}}$ to 10 and 10^3^, respectively, during the RAxML-NG bootstrapping procedure as well. According to our analyses, these changes do not affect the quality of the bootstrapping results while decreasing the runtime of the RAxML-NG bootstrap. We observe a speedup of 1.9 ± 0.8 on *Data Collection 2*.

Based on our results, the default values for $\epsilon_{\text{LnL}}$ and $\epsilon_{\text{brlen}}$ were increased in the production level release of RAxML-NG Version 1.2.0 to 10 and 10^3^, respectively. RAxML-NG Version 1.2.0 further performs the suggested tree evaluation step with conservative likelihood epsilon settings (i.e. 0.1) after each tree inference (see https://github.com/amkozlov/raxml-ng/releases/tag/1.2.0).

While our findings suggest that increasing the likelihood epsilon threshold $\epsilon_{\text{LnL}}$ to values ≥100 in both, RAxML-NG, and IQ-Tree generally yields worse results, such an increase may be (re-)considered for MSAs with specific attributes, potentially allowing for improved speedups. For future studies, we thus suggest a more thorough analysis of the impact of numerical thresholds on maximum likelihood tree inference in relation to attributes of the MSAs other than the sites-per-taxa ratio (e.g. the proportion of gaps or alternative measures/predictions of phylogenetic signal). To this end, simulating MSAs with specific attributes could constitute a way forward, albeit simulations still tend to be unrealistic (Trost *et al.* 2023). However, such a more thorough exploration should be carefully considered, as performing a vast amount of tree inferences is computationally expensive.

## Supplementary Material

vbad124_Supplementary_Data
